# Authorship revisited: Bestowing undue credit on students

**DOI:** 10.4102/sajpsychiatry.v27i0.1578

**Published:** 2021-03-10

**Authors:** Renier Steyn

**Affiliations:** 1 Graduate School of Business Leadership, University of South Africa, Pretoria, South Africa

Dear Editor

The *South African Journal of Psychiatry* (SAJP) uses the African Online Scientific Information Systems (AOSIS) platform and explicitly subscribes to AOSIS’s guidelines. African Online Scientific Information Systems, and by proxy, SAJP, states that it follows generally accepted standards regarding authorship allocation.

The particular concern I would like to raise is the AOSIS statement that, in the case of multi-authored publications, which substantially draw from a student’s academic work, the student should preferably be listed as the principal author. Such a stance affords generous credit to novice researchers and places immense responsibility on individuals now acting as senior researchers.

According to the AOSIS guidelines, the senior researcher has to guarantee the integrity of the data, assure its availability for future analyses and substantiate the methods used when the data was analysed. It would be difficult for students to fulfil this role, as they do not necessarily possess expert insight with regard to research methodology or administration. Apart from this concern, it can well be asked whether a novice researcher is able to vouch for the correctness of all aspects of the multi-authored script, which many journals require from a principal author. To complicate matters further, students often have only short-term associations with the affiliated organisation, which places the affiliated organisation at additional risk.

I suggest that it would be responsible and more equitable to embrace the stance from the American Psychology Association (APA) and accept that ‘[*e*]xcept under exceptional circumstances, a student [*should be*] listed as principal author on any multiple-authored article that is substantially based on the student’s doctoral dissertation’. I suggest that the exception should be specified, stating that the principal author (the student) is able to be ‘personally accountable for all aspects of the work, and practically assume responsibility to respond to and resolve questions related to the accuracy or integrity of the entire piece of work’. This is a very steep criterion to meet for a student, but it is fairer, balancing credit allocation with expertise and responsibility.

I am well aware that senior researchers are often willing to surrender principal authorship to students, but I judge this generous attitude as bordering on the irresponsible. This letter serves to facilitate a reflection on the practices and philosophy behind authorship allocation, as it refers to multi-authored publications based on graduate students’ work, and the SAJP stance regarding this matter.

Best,

Renier Steyn, (habitual) PhD candidate

## Acknowledgements

### Competing interests

The author declares that he has no financial or personal relationships that may have inappropriately influenced him in writing this letter.

### Author’s contribution

R.S. is the sole author of this letter.

### Ethical considerations

This letter followed all ethical standards for research without direct contact with human or animal subjects.

### Funding information

This letter received no specific grant from any funding agency in the public, commercial or not-for-profit sectors.

### Data availability

Data sharing is not applicable to this letter as no new data were created or analysed in this study.

### Disclaimer

The views and opinions expressed in this letter are those of the author and do not necessarily reflect the official policy or position of any affiliated agency of the author.

## Commentaries

### Comments on ‘Authorship revisited: Bestowing undue credit on students’

**Sean Kaliski**

Department of Psychiatry and Mental Health

University of Cape Town,

Cape Town, South Africa

Academic careers depend on publishing, and the earlier ambitious academics start, apparently the more illustrious their futures. In 1987, I attended a seminar in which Prof. Morgan of Bristol University insisted that a top academic could not produce more than two quality articles a year and decried the ‘American disease’, in which academics compulsively churned out heaps of articles. Not only has the American disease become the norm, but increasingly academics collaborate to produce multi-authored articles (as single authorship is deemed suspect for research output). Consequently, authors now jostle for the position their names occupy in the citation, because the first author is accorded the highest kudos as the acknowledged leader of the research, whereas the others’ importance diminishes as they slide down the list. And it won’t do if one’s curriculum vitae consists mostly of midlist authorships. An oft-ignored practice is the insistence of some senior researchers to have their names attached to all articles emanating from their departments, on the grounds that their minions are producing research for the senior by proxy.

Over the past four decades there has been an alarming increase in the number of published articles that have to be retracted or corrected, either because of scientific misconduct or faulty methods.^1^ Hence, as the letter points out, the principal author is not only the guarantor of the contents of the article but also may have to bear responsibility for possible misconduct. However, is this really a problem for students who are principal authors?

Virtually all high-impact journals have authorship policies, which generally embrace four criteria that each author has to satisfy, namely having made substantial contributions to the conception or design of the work, the extraction and analysis of the data, the drafting and revision of the article, as well as approval of the final version and agreement to be accountable for all aspects of the final article.^2,3,4^ Increasingly, articles are being submitted with the statement that all authors ‘contributed equally’, which seems to acknowledge that they jointly and severally are responsible for the contents. Therefore, it seems that seniors who find themselves submerged in a long list of authors cannot escape responsibility.

Clinical specialty programmes now demand that students conduct research under supervision as an essential component of their training and registration. There has also been a steady increase in the number of doctoral candidates. Most work either under close supervision or within established research teams. However, they do most of the conceptualisation of the project and the bulk of the work. Logically, they deserve the primary plaudits and are in the best position to field queries about the quality of the published article. As stated above, the senior does not escape responsibility because he or she is not the principal author. However, the student rightfully and fairly will get most of the credit. Maybe this issue is just a storm in a thimble.

### Comments on ‘Authorship revisited: Bestowing undue credit on students’

**Christopher Paul Szabo**

Honorary Professor – Department of Psychiatry

Emeritus Professor – Faculty of Health Sciences

University of the Witwatersrand Johannesburg, South Africa

The letter raises an important issue – authorship, specifically in terms of students. The apparent motivation for the letter is a concern that students may be placed in situations, through authorship, whereby they are not sufficiently equipped to take responsibility for such authorship and more specifically where they are listed as the ‘principal author’. According to the AOSIS guidelines (as noted by the letter’s author), all work that is substantially based on the work of a student should, as a published article, note the student as the principal author. The recommendation is that SAJP should adopt the approach of the APA, who, whilst also suggesting principal authorship under the same circumstances, provide for a deviation based on exceptional circumstance, which is seemingly where the student is not able to be personally responsible or accountable for all aspects of the work.

The letter’s author does not initially define the meaning of ‘students’, despite subsequently mentioning ‘graduate students’ work’. It should be noted that whilst all specialists in training are registered higher-degree students, increasingly one is seeing medical students undertake research projects as part of their undergraduate curriculum. Whether under- or post-graduate, all such work is supervised. Whilst the projects themselves serve the purpose of fulfilling certain training requirements, they should ideally also be considered for publication as articles in peer-reviewed journals. This is not necessarily a primary aim for students but may be for supervisors, whose career path may require publications to fulfil certain requirements for academic promotion. Provision is made for supervisors to proceed with publication in certain instances where after a reasonable period of time the student(s) themselves have not been inclined to do so. Under such circumstances, it could be reasonable to expect that students be included as co-authors. That being the case, student principal authorship is not an issue. However, where a student or students express the wish to be actively involved in the writing of an article, the issue of authorship is relevant and needs to be determined in advance. Under such circumstances, it is understandable that a supervisor may want to appropriately support and accord principal authorship to the student(s).

Regarding both the AOSIS and APA guidelines, it appears that neither is absolute in terms of principal authorship for students whose work substantially serves as the basis for publication, noting the letter’s author’s reference to ‘preferably’ in relation to AOSIS and the exception provided for by the APA, notwithstanding their position that a student ‘is’ to be noted as such under the aforementioned circumstance.

The key issue appears to be credit versus accountability.^5^ Alfonso et al.^5^ make reference to the four criteria required by the International Committee of Medical Journal Editors (ICMJE).^6^ Aside from the initial three criteria (which relate to the extent of contribution, drafting and final approval of an article), a fourth criterion was added in August 2013, related to being accountable for all aspects of the work with a clear understanding that all four criteria ‘… must be met by each individual author’.^5^ Alfonso et al.^5^ further elaborate on this ICMJE criterion, stating that ‘[*t*]he main novel idea is to emphasize the responsibility of each author to stand for the integrity of the entire work’.^5^ It would appear that simply ascribing principal authorship to a student on the basis of the article’s content arising substantially from the student’s work – as is recommended by both AOSIS and APA – may be too simplistic. Rather, the ICMJE criteria should be considered in serving as a basis for authorship, principal or other, which would ensure both credit and accountability.
